# LEAP-2 Counteracts Ghrelin-Induced Food Intake in a Nutrient, Growth Hormone and Age Independent Manner

**DOI:** 10.3390/cells11030324

**Published:** 2022-01-19

**Authors:** Javier Lugilde, Sabela Casado, Daniel Beiroa, Juan Cuñarro, Montserrat Garcia-Lavandeira, Clara V. Álvarez, Rubén Nogueiras, Carlos Diéguez, Sulay Tovar

**Affiliations:** 1 Departamento de Fisioloxía and Centro de Investigación en Medicina Molecular (CIMUS), Instituto de Investigaciones Sanitarias de Santiago de Compostela (IDIS), Universidade de Santiago de Compostela, 15782 Santiago de Compostela, Spain; javierlugildevalin@gmail.com (J.L.); sabela.casado.masa@usc.es (S.C.); daniel.beiroa.tarrio@usc.es (D.B.); juan.cg.1992@gmail.com (J.C.); ruben.nogueiras@usc.es (R.N.); 2 CIBER Fisiopatología de la Obesidad y Nutrición (CIBERobn), 15706 Santiago de Compostela, Spain; 3 Neoplasia & Endocrine Differentiation P0L5, Centro de Investigación en Medicina Molecular y Enfermedades Crónicas (CIMUS), University of Santiago de Compostela (USC), 15782 Santiago de Compostela, Spain; montserrat.garcia@usc.es (M.G.-L.); clara.alvarez@usc.es (C.V.Á.)

**Keywords:** LEAP-2, ghrelin, leptin, GH, adiposity, obesity

## Abstract

Data gleaned recently shows that ghrelin, a stomach derived peptide, and liver-expressed-antimicrobial peptide 2 (LEAP-2) play opposite roles on food intake. However, the data available with LEAP-2 in relation to in vivo studies are still very scanty and some key questions regarding the interplay among ghrelin and LEAP-2 remain to be answered. In this work, using rats and mice, we study fasting-induced food intake as well as testing the effect of diet exposure, e.g., standard diet and high fat diet, in terms of ghrelin-induced food intake. The anorexigenic effect of LEAP-2 on fasting induced food intake appears to be dependent on energy stores, being more evident in *ob/ob* than in wild type mice and also in animals exposed to high fat diet. On the other hand, LEAP-2 administration markedly inhibited ghrelin-induced food intake in lean, obese (*ob/ob* and DIO) mice, aged rats and GH-deficient dwarf rats. In contrast, the inhibitory effect on glucose levels can only be observed in some specific experimental models indicating that the mechanisms involved are likely to be quite different. Taken together from these data, LEAP-2 emerged as a potential candidate to be therapeutically useful in obesity.

## 1. Introduction

Ghrelin, a hormone produced in the stomach, is the most potent orexigenic hormone. Chronic ghrelin administration promotes weight gain and adiposity in rodents [1], as well as increasing voluntary food-intake in humans [2]. Assessment of circulating ghrelin levels has shown the existence of an inverse relationship with bodyweight [3], but in obese patients diagnosed with Prader–Willi syndrome increased levels of ghrelin are detected, which could explain their hyperphagia and increased body weight [4,5]. In preclinical models, loss-of-function experiments using ghrelin knockout (KO) [6] or ghrelin receptor (GHS-R1a) KO mice [7] have shown that lack of ghrelin function protects against early-onset obesity and its effects on energy balance are mediated by this receptor. Altogether it was generally acknowledged that ghrelin is a multifaceted regulator of metabolism, meaning that ghrelin regulates energy balance in the short term by the induction of appetite and in the long term by the increase in adiposity [8,9]. Of note, these effects are associated with changes in glucose and/or lipid homeostasis [10,11,12]. In general terms, it was widely accepted that all components of the ghrelin system play a key physiological role in energy homeostasis and that these key components affect the biological activity of ghrelin, ghrelin gene transcription and the acylation process [13], or changes in the expression of the GHS-R1a or its signaling pathway [14]. A game changer with this conceptual approach was provided by the recent identification of LEAP-2 as an endogenous noncompetitive ghrelin receptor antagonist [15], although some in vitro studies indicated that LEAP-2 and/or its fragments, may also act as a competitive agonist or as an inverse agonist [16]. Of note, it was shown that LEAP-2 was able to inhibit some of the main in vivo biological effects of ghrelin, such as hyperphagia, hyperglycemia and GH release in mice and rats [17]. Consistent with this, mice lacking LEAP-2 displayed enhanced ghrelin-induced feeding and GH release [18]. Thus, plasma LEAP-2/acyl-ghrelin ratio seems to be determinant to modulate GHS-R1a activity [19,20].

Although plasma LEAP-2 was positively correlated with BMI and food intake in humans and mice [21] and LEAP-2 expression was upregulated after bariatric surgery [22], the data available with LEAP-2 in relation to in vivo studies is still very scanty and some key questions regarding the interplay among ghrelin and LEAP-2 remain to be answered.

In this work, we decided to carry out a careful characterization of the in vivo effects of LEAP-2 and ghrelin on food intake and glucose homeostasis. We decided to use a variety of experimental models including rats and mice assessing fasting-induced food intake as well as testing the effect of diet exposure, standard diet and high fat diet (HFD), in terms of food intake. In mice, we studied in detail the inhibitory effect of LEAP-2, in which the orexigenic effect of ghrelin is unopposed by leptin (*ob/ob*). We also carried out a comparison of the effects of LEAP-2 in food intake in relation to NPY, the most potent orexigenic neuropeptide known to be involved in the ghrelin orexigenic signaling pathway. Since ghrelin and LEAP-2 also appear to play a dual role on GH secretion, we studied their effect on food intake in genetically GHS-R1a-deficient animals or in the context of physiological GH deficiency as aging. Finally, we also studied the effects of LEAP-2 on glucose levels. Our data showed that LEAP-2 exerted a potent anorexigenic effect in mice and rats in a variety of experimental models. In contrast, the inhibitory effect on glucose levels can only be observed in some specific experimental models indicating that the mechanisms involved are likely to be quite different. Taken together from these data LEAP-2 emerged as a potential candidate to be therapeutically useful in obesity.

## 2. Materials and Methods

### 2.1. Animals

Adult male Lewis, dwarf rats (GH-deficient rat) as described before [23] and Sprague–Dawley rats and male C57BL6J mice, male *ob/ob* mice, GHSR-KO mice (from Timo Müller Lab) were used for the experiments. For the experiments, rats and mice were maintained until 24 months of age. The mice and rats were fed with standard chow (STD) or the high fat diet (HFD) (Research Diets D12,492; 60% fat, 5.24 kcal g^−1^, Research Diets, New Brunswick, NJ, USA) for 12 weeks. The animals were housed with an artificial 12-hour light (8:00 to 20:00)/12 h dark cycle, under controlled temperature and humidity conditions and allowed free access to standard laboratory chow and tap water; animals were housed in air-conditioned rooms (22–24 °C) under a 12:12 h light/dark cycle and fed standard rat chow, or high fat diet and water *ad libitum*.

After the experiment, the mice were killed, and all tissues were rapidly explanted (WAT was weighted) and snap-frozen on dry ice. The tissues were collected and frozen at −80 °C until they were used.

The animal procedures were conducted according to the principles approved by the Animal Care Research Bioethics Committee from the University of Santiago de Compostela (license 15010/17/002); performed in compliance with the Directive 2010/63/EU and Spanish Royal Decree 53/2013, on the protection of animals used for experimental and other scientific purposes.

### 2.2. Effects of Intracerebroventricular (ICV) LEAP-2, Ghrelin and NPY Administration on Food Intake in Rats and Mice

For the central treatments, intracerebroventricular (ICV) cannulae were stereotaxically implanted under ketamine/xylazine anesthesia. Rats or mice were placed in a stereotaxic frame (*David Kopf Instruments;* Tujunga, CA, USA) under ketamine/xylazine anesthesia. The lateral ventricle was targeted using a 25-gauge needle (*Hamilton;* Reno, NV, USA) a cannula was inserted as previously described [24,25]. Animals were individually housed and used for experimentation four days later. For the central administration of ghrelin (0.45 nmol/mice or 1.5 nmol/rat), LEAP-2 (1.5 nmol/mice or 8 nmol/rat), NPY (3.5 μg/mice), or vehicle rats or mice received two injections with 10 minutes of difference between them: Vehicle-vehicle, LEAP-2-vehicle, Vehicle-Ghrelin, LEAP-2-Ghrelin.

### 2.3. Conditioned Taste Aversion

Five days before the test, mice were allowed 2 h daytime access to water. On the day of the experiment, rats (*n* = 8 rats/group) were given access to 0.15% sodium saccharin rather than water for 30 min. Immediately afterward, one experimental group was injected intraperitoneally with 0.15 mol/L lithium chloride (LiCl) in saline [24] and ICV with saline (vehicle). The second experimental group was injected intraperitoneally with saline and ICV with LEAP-2 (1.5 nmol/rat). A third experimental group (control group) was injected intraperitoneally with saline and ICV with the vehicle. Twenty-four hours later, mice were given 2 h access to a two-bottle choice test of 0.15% saccharin versus water. Data were expressed as percent saccharin preference ratio (100 × saccharin intake/[saccharin intake + water intake]).

### 2.4. Glucose Levels Measurements

Blood glucose concentration was measured with a Glucocars G+ meter (A.Menarini Diagnostics, Florence, Italy) by means of the tail vein at the different time points.

### 2.5. Hormonal and Biochemical Assays

The quantitative measurement of LEAP-2 in plasma samples was performed using a commercial enzyme-linked immunosorbent assay (ELISA) kit (Human LEAP-2 (38–77) ELISA kit, Phoenix Pharmaceuticals, Inc., Burlingame, CA, USA) according to the manufacturer’s instructions. This kit has been previously validated [21,26]. Intra-assay and inter-assay variation coefficients were <10% and <15%, respectively. The assay sensitivity limit was 0.15 ng/mL. The absorbance from each sample was measured in duplicate using a spectrophotometric microplate reader at a wavelength of 450 nm (Epoch 2 microplate reader, Biotek Instruments, Inc., Winooski, VT, USA). The results are presented as nanograms per milliliter.

### 2.6. Statistical Analysis

Values were plotted as the mean ± SEM for each genotype. To test whether the data follows a Gaussian distribution, a normality test was performed (the Kolmogorov–Smirnov test for n between 5 and 7 or the Shapiro–Wilk test for *n* > 7). For normal distributions, a parametric test was conducted; for two-group comparisons, the unpaired t-test was carried out. In the statistical analysis data with a *p*-value, less than 0.05 were considered statistically significant. For a multiple comparison test, a one-way ANOVA followed by Bonferroni’s post hoc multiple comparison test was performed. Data analysis was performed in GraphPad Prism Software Version 9.0 (GraphPad Software, San Diego, CA, USA).

## 3. Results

### 3.1. LEAP-2 Decreased Food Intake in a Dose-Dependent Manner and It Is Not Associated with Discomfort

**Figure 1:**
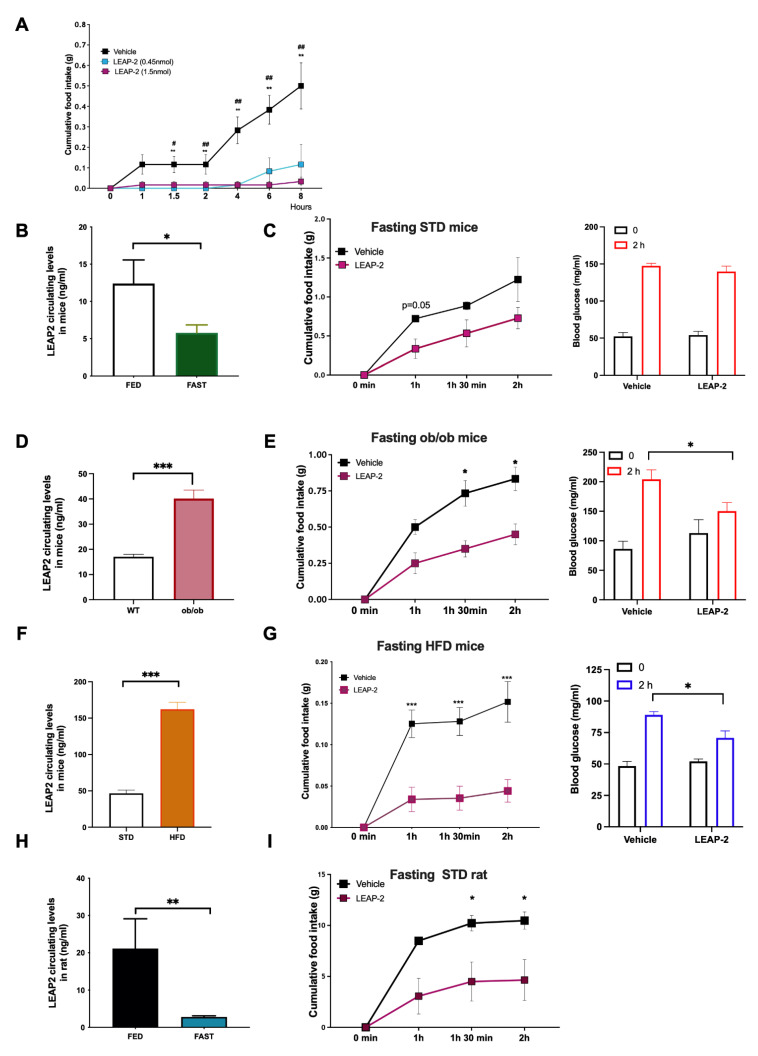
Food intake response in fasting animals after LEAP-2 administration. (**A**) Dose-response and time-response to ICV LEAP-2 administration (0.45 nmol/mice or 1.5 nmol/mice) on food intake (g) in mice. ** *p* < 0.01 between control and LEAP-2 (1.5 nmol/mice) and ^#^ *p* < 0.05, ^##^ *p* < 0.01 between control and LEAP-2 (2 μg/mice). (**B**) Circulating levels of LEAP-2 in mice in fed or under overnight fasting. (**C**) Food intake after LEAP-2 (1.5 nmol/mice) administration in mice fasting overnight (left panel) and Glucose levels before and after 2 h of LEAP-2 administration (right panel). *n* = 6. (**D**) Circulating levels of LEAP-2 in *ob/ob* mice and control. (**E**) Food intake after LEAP-2 administration in *ob/ob* mice (left panel) and Glucose levels before and after 2 h of LEAP-2 administration (right panel). *n* = 6. (**F**) Circulating levels of LEAP-2 in obese mice induced by diet (HFD) and control (**G**) Food intake after LEAP-2 administration in HFD mice fasting overnight (left panel) and Glucose levels before and after 2 h of LEAP-2 administration (right panel). *n* = 8. (**H**) Circulating levels of LEAP-2 in rats in fed or under overnight fasting. (**I**) Food intake after LEAP-2 administration in rats (8 nmol/mice) fasting overnight. *n* = 7. Data is expressed as mean ± SEM. * *p* < 0.05, ** *p* < 0.01, *** *p* < 0.001 between vehicle and LEAP-2.

To investigate if the potential central anorexigenic effect of LEAP-2 was dose-dependent we measured food intake after its single ICV administration in C57BL6 j mice fed *ad libitum* with a chow diet with two different doses: 0.45 nmol or 1.5 nmol. Both doses produced a significant decrease in food intake compared with vehicle-treated mice, with the highest dose inducing a longer suppression. (Figure 1A). Since we were planning to assess the effect of exogenous LEAP-2 in conditions of decreased circulating levels (fasting), as well as contrasting its effect upon exposure to pharmacological doses of ghrelin we decided to use the higher dose.

Firstly, we decided to rule out the possibility of any non-specific toxic effect by a standard taste-aversion test. Our data showed a similar effect between vehicle and LEAP-2 while exposure to LiCl led to a significant decrease in the saccharine preference test (Supplementary Figure S1A). A further study with this dose indicated that the effect of ICV LEAP-2 (1.5 nmol/mouse) on food intake was mediated via the GHS-R1a since contrary to the LEAP-2-induced hypophagia, we failed to obtain any effect in GHS-R1a KO animals (Supplementary Figure S1B).

### 3.2. Circulating LEAP-2 Are Modulated by Nutrient Availability and LEAP-2 Decreased Intake and Blood Glucose Levels after Fasting-Induced Refeeding

Our first objective was to figure out the interrelationship between LEAP-2 and fasting. We measured LEAP-2 circulating levels and observed a significant decrease in fasting conditions compared with ad libitum in mice and also in rats (Figure 1B,H). Next, we decided to investigate if LEAP-2 affected fasting-induced refeeding in mice. Single ICV LEAP-2 (1.5 nmol/mouse) administration produced a tendency to decrease fasting-induced hyperphagia with a chow diet (Figure 1C left panel). In fasted rats, central LEAP-2 administration significantly inhibited fasting-induced food intake (Figure 1I). Since one of the main differences between mice and rats is that the former is much more sensitive to fasting because of earlier depletion of energy stores, we decided to study mice with greater energy stores either because of obesity-induced by high fat diet or by genetic obesity (leptin deficient *ob/ob* mice). Both animal models of obesity showed higher levels of LEAP-2 in circulation in comparison to their lean controls (Figure 1D,F). When we administered LEAP-2 to *ob/ob* mice, LEAP-2 decreased the food intake stimulated by fasting (Figure 1E left panel). Similarly, in DIO mice, LEAP-2 administration also produced an inhibition of food intake (Figure 1G left panel). These results indicated that obese mice are more sensitive to the hypophagic effect of LEAP-2.

On the other hand, it is well established that ghrelin is essential for maintaining glycemia in fasting states [27]. Therefore, we next measured glucose levels after LEAP-2 administration in all the models described above. Strikingly, we found that while ICV LEAP-2 did not modify blood glucose levels after a refeeding test in lean mice (Figure 1C, right panel), in obese models (HFD and *ob/ob* mice) the glucose levels after LEAP-2 administration were lower than in control mice after refeeding (Figure 1E,G right panel). These data suggest that central LEAP-2 can reduce both feeding and glucose levels after fasting-induced refeeding.

### 3.3. Central LEAP-2 Antagonizes Ghrelin-Induced Food Intake in a Leptin- and Other Orexigenic Signals-Independent Way

**Figure 2:**
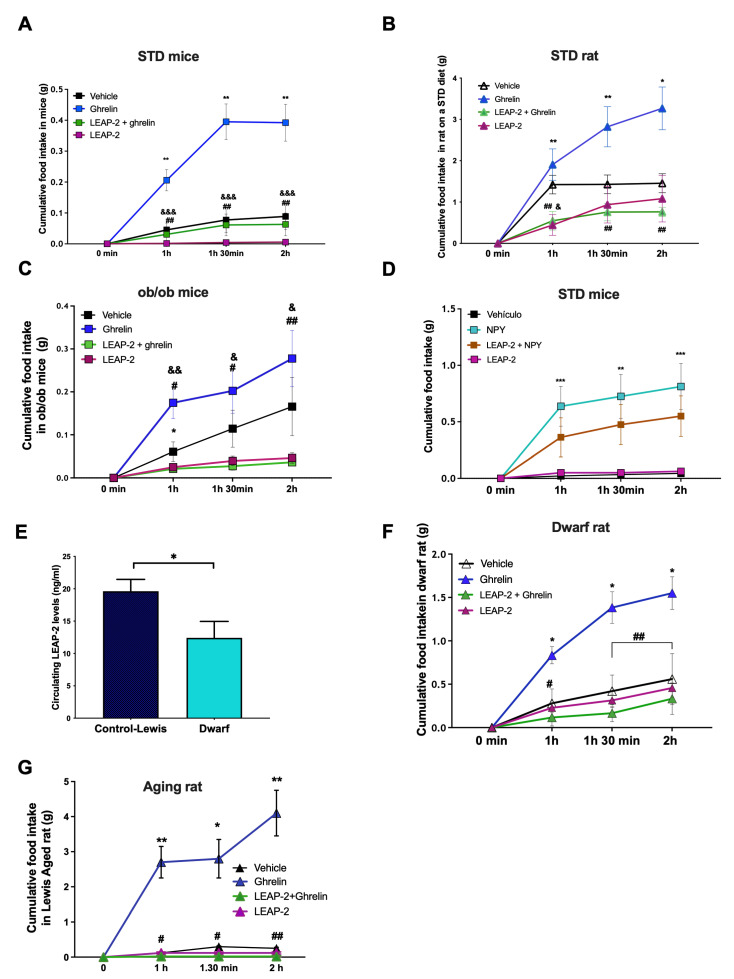
LEAP-2 decreased food intake and antagonize ghrelin effect independently of leptin and NPY. (**A**) Response to ghrelin (0.45 nmol/mice) effect in food intake during 2 h in mice treated previously with LEAP-2 (1.5 nmol/mice) ICV or vehicle on a STD diet. *n* = 8 (**B**) Response to ghrelin (1.5 nmol/rat) effect in food intake during 2 h in rats treated previously with LEAP-2 (8 nmol/rat) or vehicle ICV in rats on a STD diet. *n* = 8. (**C**) *ob/ob* mice. *n* = 6. (**D**) Response to NPY (3.5 μg /mice) on food intake during 2 h in mice treated previously with LEAP-2 (1.5 nmol/mice) ICV or vehicle in mice on a STD diet. *n* = 8. (**E**) LEAP-2 circulating levels in dwarf rats. (**F**) Response to ghrelin (1.5 nmol/rat) effect in food intake during 2 hours in dwarf rats treated previously with LEAP-2 (8 nmol/rat) ICV. *n* = 6. (**G**) Response to ghrelin (1.5 nmol/rat) effect in food intake during 2 hours in Lewis aging rats treated previously with LEAP-2 (8 nmol/rat) ICV in rats on a STD diet. *n* = 7. Data is expressed as mean ± SEM. * *p* < 0.05, ** *p* < 0.01, *** *p* < 0.001 between vehicle and ghrelin; ^#^ *p* < 0.05, ^##^ *p* < 0.01 between ghrelin and LEAP-2 + ghrelin; ^&^ *p* < 0.05, ^&&^ *p* < 0.01, ^&&&^ *p* < 0.001 between vehicle and LEAP-2.

Next, we proposed to figure out if LEAP-2 could antagonize the orexigenic effects of ghrelin. The ICV co-administration of LEAP-2 and ghrelin in fed *ad libitum* mice produced a clear reduction of ghrelin-stimulated food intake (Figure 2A) and rats (Figure 2B) after 60, 90 and 120 min. In agreement with our previous findings (Figure 1A), the administration of ICV LEAP-2 alone produced a decrease in food intake with values significantly smaller than control mice.

Leptin is a well-known anorexigenic hormone with opposing effects to ghrelin in food intake and adiposity. Thus, we decided to assess the anorexigenic effect of LEAP-2 in the absence of leptin but under pharmacological ghrelin stimulation. The administration of LEAP-2 in *ob/ob* mice decreases food intake compared with the vehicle (Figure 2C). As expected, ICV ghrelin stimulated feeding after 2h, but the co-administration of LEAP-2 with ghrelin antagonized the orexigenic effect of ghrelin (Figure 2C). This suggests that ghrelin and LEAP-2 can modulate food intake and that their counteracting effects are leptin-independent.

The orexigenic activity of ghrelin is mediated by NPY, a potent neuropeptide that increases food intake. In order to investigate if the anorectic effect of LEAP-2 could be mediated by NPY, we co-administered ICV LEAP-2 and NPY to mice fed a chow diet (Figure 2D). As expected, NPY produced a clear increase in food intake compared with vehicle-treated mice, and mice co-treated with LEAP-2 and NPY showed a tendency to decrease food intake that did not reach statistical significance (Figure 2D).

One of the main features of ghrelin and LEAP-2 is that they have opposite effects on GH secretion [15]. Therefore, we decided to assess their effects on food intake in GH deficient animals, namely dwarf rats. First, we observed that circulating LEAP-2 levels were lower in dwarf rats compared with control rats (Figure 2E). Then we found that ICV LEAP-2 elicited a clear-cut inhibition of ghrelin induced-food intake (Figure 2F). In addition, animals receiving LEAP-2 after fasting exhibited a decrease in food intake in comparison to vehicle treated animals without changes in glucose levels (Supplementary Figure S1C). Finally, we studied a model of physiological GH deficiency, namely the aging rat. We found a clear-cut stimulatory effect of ghrelin in aged rats that was almost completely blunted by LEAP-2 (Figure 2G). Overall, these data indicate that the opposite interaction between LEAP-2 and ghrelin is not dependent on GH levels.

## 4. Discussion

Following the seminal discoveries of leptin and ghrelin, it was generally accepted that they play a key role in food intake as the signals influence satiety and hunger, and therefore, dictate whether to eat or not to eat. Furthermore, it was shown that in terms of energy homeostasis this interaction takes place mainly in the arcuate nucleus of the hypothalamus [9]. In this context, the discovery of LEAP-2 as an endogenous non-competitive ghrelin receptor antagonist [15] raised many expectations, as well as many unanswered questions. So far, available in vivo data is quite scarce [15,17,18,21] hence our aim in providing new knowledge with particular emphasis on the assessment of acute food intake. As a whole, we provide strong evidence that central exogenous LEAP-2 administration exerts a marked anorexigenic effect in three different rodent animal models (lean, obese and aged) and also in a variety of experimental models.

We first assessed the effect of LEAP-2 in fasting-induced food intake in rats and mice because fasting is associated with decreased leptin and increased ghrelin levels. The finding that fasting decreased LEAP-2 levels is in agreement with previous studies [21]. Our finding showing a lack of clear effect of exogenous LEAP-2 on fasting-induced food intake was compatible with several non-mutually exclusive possibilities. In the context of a low leptin tone, other anorexigenic signals will be unable to exert a meaningful effect, generating a fasting-induced state of LEAP-2 resistance, a phenomenon related perhaps to mice developing specific mechanisms to adapt their response to fasting given their low energy stores. Our data showing that the anorexigenic effect was present in fasting rats support the last one. Our data is in contrast to the one obtained by others [17] in rats, the differences likely related to the lower dose of LEAP-2 used in their study. In keeping with our data in rats, in obese mice, which have larger energy stores, under similar fasting conditions we observed a clear-cut feeding inhibitory effect of LEAP-2. Of note, the anorexigenic effect of LEAP-2 observed in mice exposed to HFD is in contrast to the leptin resistance usually observed in diet induced obesity. Additionally, our data showing that LEAP-2 was able to suppress food intake in *ob/ob* mice is quite interesting since it shows that its effect is independent of endogenous leptin tone raising the possibility of LEAP-2 based drugs as potential candidates for management of leptin deficiency or leptin resistant related obesity.

The data obtained on fasting-induced food intake was suggestive of LEAP-2 inhibiting ghrelin orexigenic effect, although there are many others changes during fasting in other relevant signals, such as leptin and GH, that should not be ignored. Therefore, we tested the effect of LEAP-2 following exogenous ghrelin administration in different experimental models. Our data showing that LEAP-2 inhibits ghrelin orexigenic effect in mice and rats are in agreement with previous findings [15]. Our data showing a clear effect of LEAP-2 on ghrelin induced food intake in *ob/ob* mice are quite interesting as it was shown that genetic deletion of ghrelin [28] or the ghrelin-receptor gene in *ob/ob* mice are unable to reverse their hyperphagia [29]. Taking into account that LEAP-2 is targeting the GHSR, the data look contradictory at first glance. However, it is likely that these discrepancies are due to compensatory phenomena elicited by genetic deletion. In keeping with our findings, a previous study using a synthetic GHR-antagonist also reported a decrease in food intake [30]. In line with our data in *ob/ob* mice, we also found an anorexigenic effect of LEAP-2 in ghrelin-induced food intake in another obesity model, the DIO mice, which is in clear contrast to the leptin-resistant state observed in DIO mice and highlights its potential translational relevance.

Data gleaned over the last few years have shown that ghrelin orexigenic signal is mediated to a large extent by the AMPK signaling pathway dependent on NPY/AgRP neurons [9,31,32]. Thus, we decided to assess whether LEAP-2 could influence the orexigenic effect exerted by a downstream ghrelin signal as NPY. As expected NPY exerted a powerful orexigenic effect that was partially inhibited by exogenous LEAP-2 administration in contrast to the expected full suppression. However, it should be noted the existence of alternative pathways to NPY is a potential explanation of the lack of full expression [8,9,33]. Further work with more molecular studies will be needed to test the implication of LEAP-2 in specific ghrelin signaling pathways like p53-Sirt1-AMPK-CPT1c-BSX-NPY/AgRP pathway [32,34,35,36], the mTOR pathway [37] and a kappa-opioid receptor pathway [33] and uncovering the molecular pathways involved in the LEAP-2 actions.

In addition to its known actions in lipid and glucose homeostasis, GH also influences food intake by acting at a central level where GH receptors (GHR) are highly expressed. The interplay between ghrelin, growth hormone and energy homeostasis is also supported by data showing that ghrelin orexigenic effect is at least partially mediated by central GHR, being blunted in animals with genetic deletion of the GHR [38]. Taking into account that LEAP-2 inhibits GH secretion, it was theoretically possible that lack of GH and consequent activation of the central GHR could be mediating the LEAP-2 anorexigenic effect. Our data showing that in dwarf rats, with very low circulating GH levels, the anorexigenic effect of ghrelin is still present argues against that possibility. This data is further supported by similar effects observed in aged animals, in which GH secretion is also known to be decreased [39]. We found that LEAP-2 led to a clear-cut decrease in ghrelin orexigenic effect. In fact, it almost completely blunted the orexigenic effect of ghrelin. Further work comparing lower doses of LEAP-2 in young and old animals is needed in order to test the possibility of an age-related increase in sensitivity to LEAP-2.

As we described before, ghrelin is essential in the counterregulatory action of glucose, increasing blood glucose levels. Therefore, we would expect that LEAP-2 maintains low blood levels and indeed this result was obtained in mice but only under HFD. Additionally, in monogenetic obese model *ob/ob* mice, LEAP-2 administration produced lower glucose blood levels than mice treated with vehicle after 2 hours refeed.

The relevant role of ghrelin in glucose homeostasis is beyond any doubt. These effects are exerted, both, by acting in peripheral tissues and in the brain by a variety of mechanisms. At the periphery, they include pancreatic hormone secretion, gastrointestinal mobility, intestinal glucose adsorption, glucose metabolism at peripheral tissues or GH and cortisol secretion [40]. In the brain, where ghrelin receptors are widely distributed, it was shown to exert multiple effects through the autonomous nervous system including regulation of pancreatic hormone secretion. These many effects of ghrelin could explain some doubts regarding its effects on glucose in fed animals as shown in *ob/ob* animals where genetic deletion of ghrelin resulted in marked improvement in the hyperglycemia produced for leptin-deficiency while deletion of GHSR had the opposite effect, it worsened the hyperglycemia of the *ob/ob* mice [29]. In contrast, it is now widely accepted that its main role is likely related to states of negative energy balance where ghrelin prevents hypoglycemia. Our data showing some discrepancies of LEAP-2 regarding food intake and glucose levels were, therefore, not unexpected. It was previously shown that controlled re-expression of physiological levels of ghrelin receptors (GHSRs) in specific neuronal populations means their glucose and orexigenic responses can be dissociated [41,42]. On the other hand, the reasons for our findings that the effect of LEAP-2 on glucose levels was only observed in obese animals is yet unclear in mechanistic terms. It may be possible that obese animals exhibit a state of hyperglycemia and insulin resistance, and therefore, a higher set point to elicit a counterregulatory response to prevent hypoglycemia following LEAP-2 administration. Future studies using the glucose clamp technique are needed to answer this question which may also help to foresee its potential use in obesity and type 2 diabetes.

In sum, we extend previous findings regarding the relevance of LEAP-2 as an anorexigenic signal. Specifically, our data using different experimental models: lean, obese (*ob/ob* and DIO), aged rats and GH-deficient rats shows that LEAP-2 is a potent anorexigenic agent and maintains its capacity to blunt ghrelin-induced hyperphagia. Further work assessing its effects following chronic administration is merited in order to establish its potential in terms of drug development.

## Data Availability

Not applicable.

## Supplementary Materials

The following supporting information can be downloaded at: https://www.mdpi.com/article/10.3390/cells11030324/s1, Figure S1: LEAP-2 does not produce toxic effects and food intake action is mediated via the GHS-R1a.
