# Supplementary material for: LEAP-2 Counteracts Ghrelin-Induced Food Intake in a Nutrient, Growth Hormone and Age Independent Manner
Source: Cells. 2022 Jan 19;11(3):324. doi: 10.3390/cells11030324 (PMC8834077; doi:10.3390/cells11030324)
Supplement: Supplementary file 1 [file cells-11-00324-s001.zip › cells-1551463-supplementary.pdf]

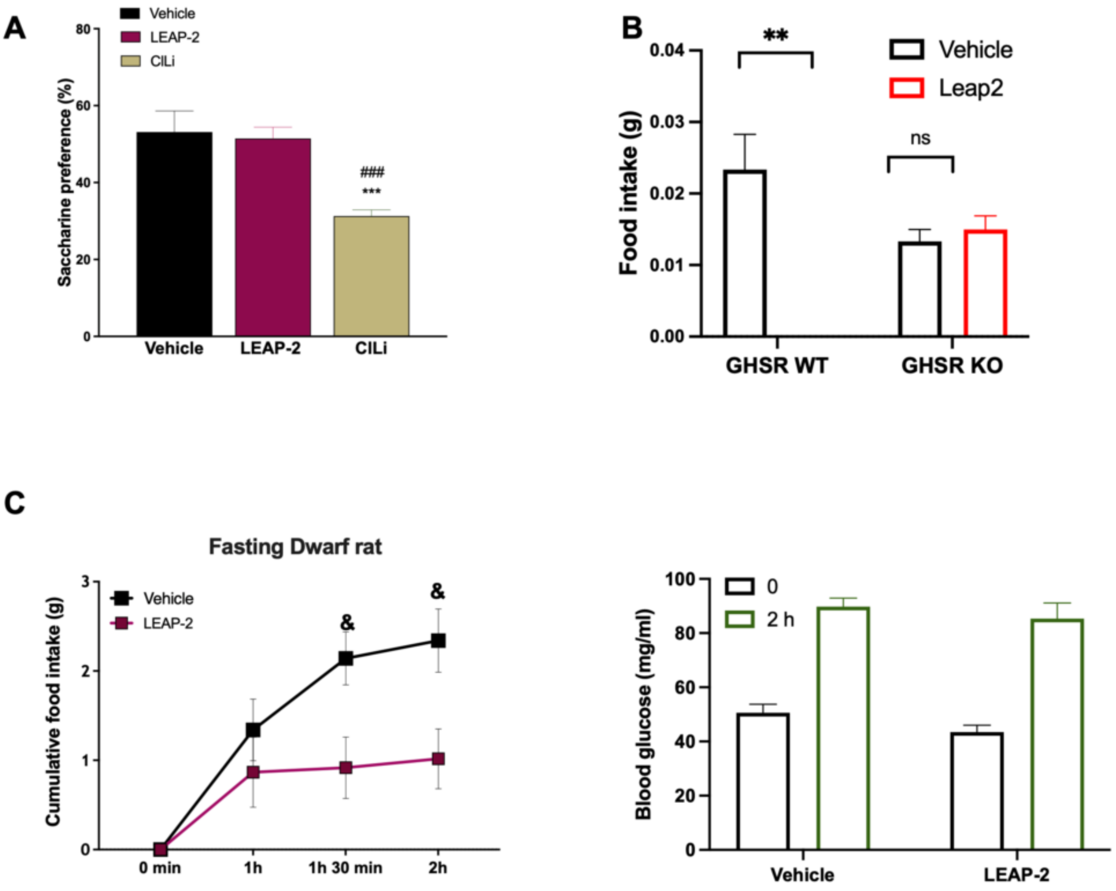

Figure S1. LEAP-2 does not produce toxic effects and food intake action is mediated via the GHS-R1a. (**A**) Conditional aversion test in mice treated with LEAP-2 (1.5 nmol/mice) or vehicle ICV. $n = 8$. (**B**) Food intake 2 hours after LEAP-2 administration in GHSR KO and wt mice. $n = 9$. (**C**) Food intake after LEAP-2 administration in dwarf rats fasting overnight (left panel) and Glucose levels before and after 2 h of LEAP-2 administration (right panel). n = 6. Data is expressed as mean ±SEM. ** $p < 0.01$, *** $p < 0.001$ between vehicle and LEAP-2. $^{###}$ $p < 0.001$, $^{\&}$ $p < 0.05$ between vehicle and ClLi.
